# Hematopoietic stem cell transplantation as a curative treatment for Nck-associated protein 1-like (HEM1) deficiency: A first case report

**DOI:** 10.70962/jhi.20250228

**Published:** 2026-03-17

**Authors:** Julian Thalhammer, Bénédicte Bruno, Sylvain Latour, Benjamin Fournier, Wadih Abou Chahla

**Affiliations:** 1 Department of Pediatric Hematology, Hôpital Jeanne de Flandre, CHU de Lille, Lille, France; 2 Laboratory of Lymphocyte Activation and Susceptibility to EBV Infection, INSERM UMR 1163, Imagine Institute, Université de Paris, Paris, France; 3 Department of Pediatric Immunology, Hematology and Rheumatology, Necker Enfants Malades Hospital, Assistance Publique-Hôpitaux de Paris, Paris, France

## Abstract

We report two siblings with hematopoietic protein 1 (HEM1), also named Nck-associated protein 1-like (NCKAP1L), deficiency and varying clinical phenotype and the first case of successful hematopoietic stem cell transplantation for recurrent infections and EBV-induced hemophagocytic lymphohistiocytosis, showing curative potential.

## Teaser

Thalhammer et al. report two siblings with hematopoietic protein 1 (HEM1), also named Nck-associated protein 1-like (NCKAP1L), deficiency and varying clinical phenotype and the first case of successful hematopoietic stem cell transplantation for recurrent infections and EBV-induced hemophagocytic lymphohistiocytosis, showing curative potential.

We report on two siblings with hematopoietic protein 1 (HEM1), also named Nck-associated protein 1-like (NCKAP1L), deficiency and the first case of successful hematopoietic stem cell transplantation (HSCT). HEM1 is a part of the WAVE regulatory complex, and mutations are linked to an inborn error of immunity (IEI) with the hallmarks of a combined immunodeficiency such as recurrent bacterial and viral infections, autoimmunity, autoinflammation, and hemophagocytic lymphohistiocytosis (HLH) (1, 2, 3). The WAVE regulatory complex plays an important role in the regulation of actin networks. So-called actinopathies are an emerging field of IEI with Wiskott-Aldrich syndrome (WAS) being the most prominent disease but also DOCK8 and 11 deficiencies (4). The cytoskeletal structure is important for the movement, interaction of immune cells, and the formation of the immunological synapse. HSCT is the standard of care and curative for WAS, and there is extensive experience with DOCK8 deficiency, showing an overall survival rate of 80–90%. However, for HSCT in rarer actinopathies, only case reports or small cohorts have been published, and no experience with HEM1 deficiency exists so far.

**Figure 1.**
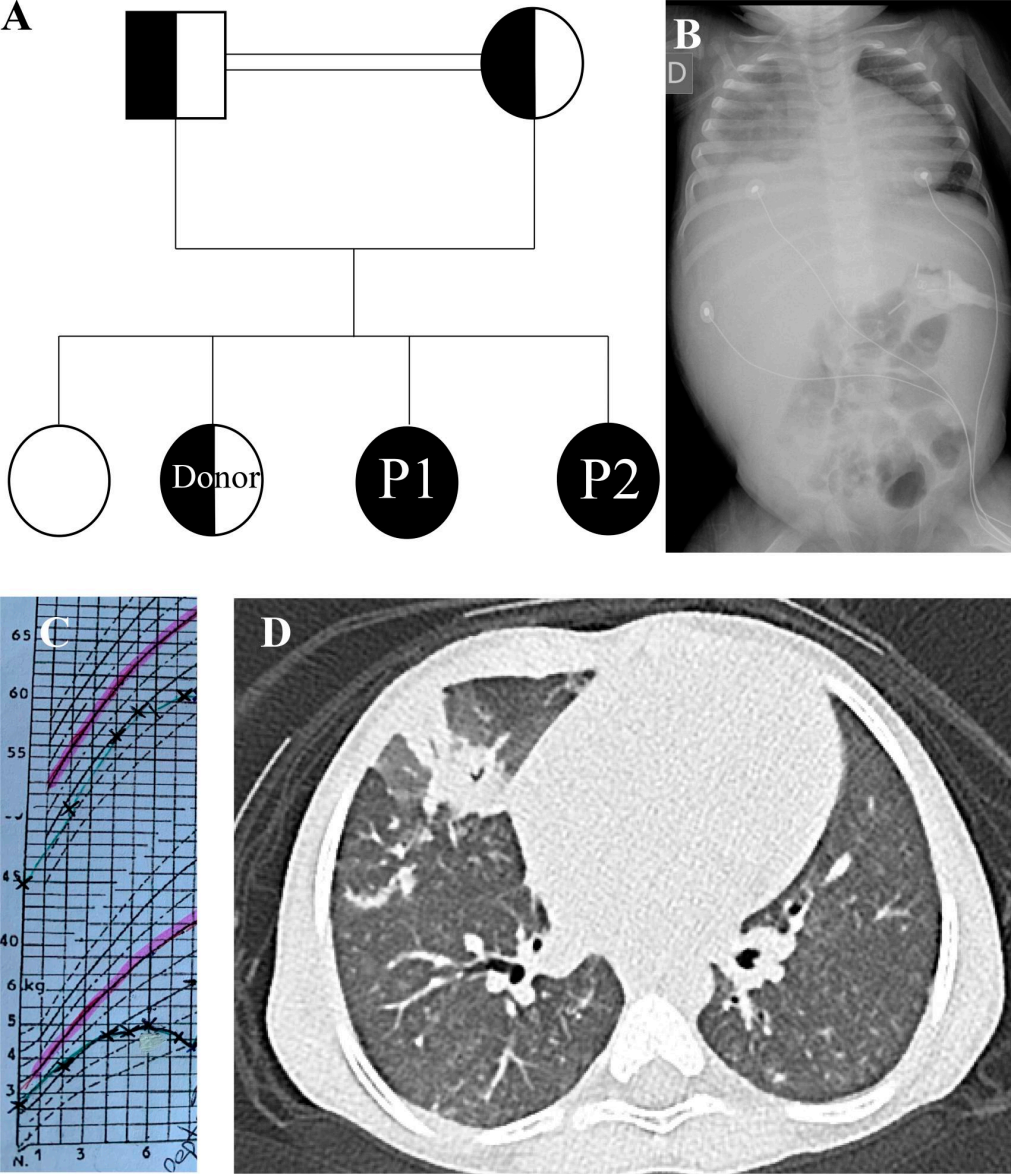
**Clinical details of the patients with a pedigree, images and percentiles** **. (A)** Pedigree of the patient family with a homozygous NCKAP1L mutation identified in both affected children P1 and P2.** (B)** X-ray with massive hepatosplenomegaly at the moment of HLH. **(C)** Extract of the child’s health record booklet with the percentiles of height (upper curve) and weight (lower curve). **(D)** Chest CT scan with nodular infiltrations.

We report on the third and fourth child of consanguineous parents with HEM1 deficiency (Fig. 1 A). Clinical details for the older patient 1 have been reported previously by Cook et al. in 2020 (patient 4.1), whereas her younger sister is newly described here.

Patient 1 exhibited intrauterine growth retardation, and in the first year of life, she suffered from multiple infectious episodes, including severe respiratory syncytial virus,, several pulmonary infections, an episode of sepsis associated with otitis, gastroenteritis, and pyelonephritis. At 10 mo of age, she had gastrostomy due to failure to thrive (Fig. 1 C). At 12 mo, she experienced a severe episode of HLH triggered by EBV infection, requiring pediatric intensive care unit and hospitalization for 3 mo (Fig. 1 B). HLH was controlled by corticosteroids, rituximab, and long-term cyclosporine A maintenance. Intravenous immunoglobulin replacement was started at 2.5 years of age for recurrent infections despite normogammaglobulinemia. Still, she continued to experience recurrent bronchitis and otitis even on prophylactic cotrimoxazole. A homozygous disease-causing mutation in *NCKAP1L* was identified at 3 years of age: c.773G>7; p.Arg258Leu. No other pathological mutation was identified in the exome analysis.

At 5 years, we proceeded with HSCT given the severity of the disease course. Pretransplant evaluation revealed failure to thrive (height and weight at the first percentile), chronic diarrhea, clinical hepatosplenomegaly (10 cm), chronic serous otitis, persistent respiratory congestion, and neurodevelopmental delay. She had persistent positive PCR for EBV in the blood with 4.4 log10, lungs with 4.3 log10, and 5.0 log10 on a stomach biopsy. Chest computerized tomography (CT) demonstrated both macro- and micronodular infectious lesions, adenomegaly, and bronchial dilation (Fig. 1 D). She suffered from chronic nasal obstruction with chronic serous otitis with impaired hearing. Paracentesis with implantation of grommets, adenoidectomy and amygdalotomy normalized hearing and breathing. She was in remission of her HLH with cyclosporine A maintenance without flares, but persistent hepatosplenomegaly. Liver biopsy showed periportal and perisinusoidal fibrosis (F1). Transaminases were slightly elevated. Endoscopy revealed aphthous ulcerations, follicular hyperplasia, and signs of digestive tract inflammation. Inflammatory bowel disease as an autoimmune manifestation of her IEI was suspected. Brain magnetic resonance imaging showed cortical gyration abnormalities, left-sided ventriculomegaly, and periventricular heterotopias. Echocardiography showed normal cardiac function and a bicuspid aortic valve.

She underwent HSCT from a healthy HLA-matched sibling, heterozygous for NCKAP1L (Fig. 1 A), blood group matched, and seropositive for CMV and EBV*.* Rituximab treatment was intensified to ablate EBV before HSCT and serotherapy with alemtuzumab were given in the context of previous HLH. She received myeloablative conditioning according to European Society for Immunodeficiencies working party guidelines with fludarabine 150 mg/m^2^ (d-7–d-3, 30 mg/m^2^/day), treosulfan 42 g/m^2^ (d-6–d-4, 14 g/m^2^/day), thiotepa 10 mg/kg on d-2, alemtuzumab 0.9 mg/kg (d-16–d-14, 0.3 mg/kg/day), and rituximab 750 mg/m^2^ (d-26 and d-21, 375 mg/m^2^) (5). She received a bone marrow graft with 7.42 × 10^6^/kg CD34^+^ cells and 5.88 × 10^8^/kg nucleated cells. EBV PCR was negative throughout the whole HSCT. She had hyperthermia without microbiological documentation and no mucositis. She had severe grade III sinusoidal obstruction syndrome treated by defibrotide and several days of oxygen support of maximum 1 L/min. Neutrophils were engrafted on d21, and platelets were engrafted on d33. She had no graft-versus-host disease (GVHD). Cyclosporine A was tapered until 1.5 years after HSCT. 4 years after HSCT, the patient is well, without immunosuppressive treatment, GVHD, or need for nutritional support. Immunophenotype has normalized with complete chimerism. Lymphocyte subsets normalized apart from mild NK-penia after HSCT. Colitis normalized and hepatic fibrosis stabilized. Splenomegaly is still palpable at 5 cm, but no adenopathy. She is now 9 years old and going to school with an assistant and shows developmental progression but still has a neurodevelopmental delay in expressive language, concentration difficulties, and motor skills. HSCT did not have a significant effect on weight and height, which progress, but remain between the first and the third percentile.

Patient 2 is now 5 years old, and genetic diagnosis recently confirmed the same homozygotic mutation in *NCKAP1L*. She presents with recurrent upper and lower respiratory and ear infections, for which she received paracentesis and grommets. Her chest CT shows postinfectious changes and mediastinal adenopathy. She has persistent positive PCR EBV around 4.0 log10, splenomegaly, hypergammaglobulinemia with IgG at 23 g/L, low-normal IgM and reduced and decreasing IgA, normal vaccination responses after a booster vaccination, and low naïve T cell populations with 25 % CD4^+^ and 16% CD8^+^. B cell subpopulations were normal. She has iron deficiency, but no failure to thrive and is following the 25th percentile. Of note, she does not present any frank neurological changes, liver disease, HLH, dysimmunity, or malformations. She is better since start of cotrimoxazole and azithromycin prophylaxis. In our opinion, HSCT is not indicated at the moment, but we would discuss HSCT with the family if she presents with HLH or infections persist despite antibiotic prophylaxis and immunoglobulin replacement.

Regarding her immunological phenotype, patient 1 had, initially, in her first year normal immunoglobulin levels, but low levels of polio and tetanus vaccine serologies. At 5 years, her lymphocyte subsets showed normal counts for total CD3, CD4, CD8, and B and natural killer (NK) cells but decreased CD4^+^ naive T cells (CD4^+^, CCR7^+^, and CD45RA^+^) with 20% and 309/mm^3^ and decreased CD8^+^ naive T cells (CD8^+^, CCR7^+^, and CD45RA^+^) with 20% and 345/mm^3^, and B cell subpopulations 4 years after rituximab were remarkable for decreased switched memory B cells (CD19^+^, CD27^+^, and IgD^−^) with 0.5% of CD19 cells (2/mm^3^) and in parallel high transitional B cells (CD19^+^, CD38High, and IgMHigh) with 18% of naïve B cells (87/mm^3^) and high CD19^+^, CD38^−^, and CD21Low B cells with 52% of naïve B cells (250/mm^3^). She had no IgA production, but normal IgM and IgG under immunoglobulin supplementation.

In summary, our study reveals a distinct immunological phenotype in two siblings with HEM1 deficiency, although their clinical courses have differed. The variable degrees of hyperinflammation and HLH are present in other IEI as well. Of note, the neurological phenotype, the malformations, and failure to thrive seen in patient 1 cannot be attributed with certainty to her HEM1 deficiency. In the context of consanguinity, it cannot be excluded that there is another genetic disease, responsible for the more syndromic phenotype in patient 1, even if no other genetic aberration was found on a trio exome analysis. Cook et al. mention 2/9 patients with syndromic features and only our patient 1 with neurological abnormalities. A role for HEM1 is described in bone metabolism and cell migration. With a widening of the phenotype, more patients may possibly present syndromic features or malformations.

## Human experimental guidelines approval

This study is a retrospective case report without intervention and does therefore not require approval of an ethics committee.

## Data Availability

The data are available from the corresponding author upon reasonable request.
